# Data-driven leisure-time physical activity trajectories of over 46 years and their associations with cognition in nonagenarians: a cohort study

**DOI:** 10.1093/geronb/gbag065

**Published:** 2026-04-09

**Authors:** Paula Iso-Markku, Gabin Drouard, Vahid Farrahi, Anni Varjonen, Henri Vähä-Ypyä, Tommi Vasankari, Jaakko Kaprio, Eero Vuoksimaa, Sari Aaltonen

**Affiliations:** Institute for Molecular Medicine Finland FIMM, University of Helsinki, Helsinki, Finland; Clinical Physiology and Nuclear Medicine, University of Helsinki and Helsinki University Hospital, Helsinki, Finland; Institute for Molecular Medicine Finland FIMM, University of Helsinki, Helsinki, Finland; Research Group of Data Analytics in Sport Sciences, Institute for Sports and Sport Sciences, TU Dortmund University, Dortmund, Germany; Institute for Molecular Medicine Finland FIMM, University of Helsinki, Helsinki, Finland; The UKK Institute for Health Promotion Research, Tampere, Finland; The UKK Institute for Health Promotion Research, Tampere, Finland; Faculty of Medicine and Health Technology, Tampere University, Tampere, Finland; Institute for Molecular Medicine Finland FIMM, University of Helsinki, Helsinki, Finland; Institute for Molecular Medicine Finland FIMM, University of Helsinki, Helsinki, Finland; Institute for Molecular Medicine Finland FIMM, University of Helsinki, Helsinki, Finland; (Psychological Sciences Section)

**Keywords:** Physical exercise, Cognitive function, Episodic memory, Semantic fluency, Longitudinal

## Abstract

**Objectives:**

In short follow-up studies, greater leisure-time physical activity (LTPA) has been associated with better cognition in old age, but more longitudinal studies are needed. Our aim was to identify long-term LTPA trajectories from midlife to late old age and examine whether these trajectories are associated with nonagenarians’ cognition.

**Methods:**

In total, 125 participants from the NONAGINTA—Memory and Health in Nonagenarians study were included. The participants responded to health surveys of the older Finnish Twin Cohort study, including LTPA at the mean ages of 45, 52, 59, and 91 years. Cognition was assessed at the mean age of 91 years (standard deviation 1.54) via telephone interview (global cognitive function, episodic memory, and semantic fluency). We identified LTPA trajectories with K-means clustering for longitudinal data, and we used generalized estimating equations models to investigate differences in cognition among the LTPA trajectories. Covariates included age, sex, and education.

**Results:**

We found 3 LTPA trajectories from midlife to nonagenarian age. The largest proportion of participants belonged to the *Constant low* trajectory (52%), characterized by a stable low level of physical activity throughout the follow-up. Two other trajectories were *Starting low and increasing* (25%) and *Starting high and decreasing* (23%). Nonagenarians’ cognitive measures did not differ among the LTPA trajectories.

**Discussion:**

Longitudinal physical activity behavior may not preserve cognitive function in those who survive to nonagenarian age but larger studies are warranted.

Modifiable lifestyles have great potential in the prevention of dementia. In previous meta-analyses, physical activity has been associated with less cognitive decline in late life and a lower incidence of dementia (Iso-Markku et al., 2022b, 2024). Causal evidence on the topic is lacking (Ciria et al., 2023), but the challenges of randomized controlled trials remain to be sufficient compliance and sufficient length, especially when considering a disease with an exceptionally long pre-clinical phase. Observational studies can provide long-term or even life-course information on the possible protective factors of dementia, while avoiding the problem of poor compliance.

To date, most observational studies published on the association between physical activity and dementia or cognitive decline rely on physical activity measurements at a single time point (Iso-Markku et al., 2022b, 2024). However, examining the association between longitudinal physical activity patterns and cognition would provide a more in-depth perspective into this association since the magnitude, rate, and timing of physical activity changes can then be considered. So far, only a few existing studies on physical activity and cognitive functioning have utilized longitudinal physical activity trajectory study designs. For example, Sabia et al. (2017) found that physical activity trajectories start to decline nine years before dementia diagnosis. This may be accounted for by reverse causation, that is, the effect of subclinical disease on the ability to exercise. Previous studies on longitudinal physical activity trajectories have also shown that those participants whose cognitive performance declines at ages 60–75 most likely belong to the low physical activity trajectories (Li et al., 2022; Wagner et al., 2020) or that physical activity trajectories with a declining trend precede (Hu et al., 2022) or are concurrent with cognitive decline (Cheval et al., 2021; Wang et al., 2024). A recent study of Chinese women showed that it is not individuals who belonged to the highest or lowest physical activity trajectories, but rather to the most stable physical activity trajectory that performs best cognitively (Gong & Baek, 2025). In their study, the “Moderate—Increasing” physical activity trajectory was significantly associated with global cognitive function and episodic memory only in women aged 50- to 59-year old (Gong & Baek, 2025). In a study cohort of individuals under age 45, consistently high physical activity trajectories were shown to be associated with only one cognitive test: that is, better verbal fluency out of eight cognitive tests altogether (Gafni et al., 2022).

The participants of most previous studies investigating the association between physical activity trajectories and cognition have been older adults aged 70 to 80 years at the time of cognition assessment (see Supplementary Figure 1). Nevertheless, older adults aged 80 years and over are a rapidly growing age group, predicted to triple between 2015 and 2050 (He et al., 2016). Even among older adults aged 85 years and over, nonagenarians are a unique group. Only a few studies have explored the association of preceding physical activity and cognition among nonagenarians, with all population-based studies showing no association (Gao et al., 2017; Gow et al., 2017; Strozza et al., 2020). Only the one volunteer-based study on the subject found a significant association between high physical activity levels and better subsequent cognition in nonagenarian women (Sumic et al., 2007). However, to our knowledge, no nonagenarian study has investigated the association between physical activity and cognition considering physical activity behavior from several time points over multiple decades.

Our aim was to identify 46-year leisure-time physical activity (LTPA) trajectories based on self-reports from midlife to the tenth decade of life and examine whether cognition in nonagenarians is associated with the LTPA trajectories identified. We used data-driven methods that enable finding patterns within the data without any influence of a priori assumptions. LTPA denotes commuting and other LTPA in this study, in contrast to occupational physical activity, which has a differential association with health than LTPA (Iso-Markku et al., 2022b). Earlier, we validated the nonagenarians’ self-reported physical activity against contemporary accelerometer-based physical activity (Aaltonen et al., 2023). In this study, we further examine the association between nonagenarians’ self-reported physical activity and 46-year LTPA trajectories.

## Methods

The participants of the current study were from the longitudinal population-based data set of the Older Finnish Twin Cohort study launched in 1975 (Kaprio et al., 2019). The cohort consists of twins from same-sex pairs born in Finland before 1958. The participants have responded to the mailed health and behavior surveys in 1975, 1981, and 1990 (response rates 77%–89%, Supplementary Figure 2). In the current study, we used data from all three surveys. Health survey and cognitive interview data from the NONAGINTA—Memory and Health in Nonagenarians (response rate 29%) substudy of all twins who reached 90 years of age was conducted in 2020–2024 and defined as the fourth and latest follow-up time point (Aaltonen et al., 2023).

To create longitudinal LTPA trajectories from midlife to the tenth decade of life, we included those twins in the study who had LTPA data and were between ages 42 and 51 (mean: 45.2 years) at baseline. In 1981 and 1990, the participants were at the mean ages of 51.6 years (range: 48–58 years) and 59.2 years (range: 57–60 years), respectively. At the last follow-up time point, the participating twins had reached the mean age of 91.2 years (range: 90–97 years). Hereafter, the mean ages are referred to as 45, 52, 59, and 91. In total, we had LTPA data from at least two time points available from 125 twin individuals (65% women), including 14 complete twin pairs: 107 individuals at age 45, 112 individuals at age 52, 66 individuals at age 59, and 125 individuals at age 91. Most participants had LTPA data from three or four time points (106 participants, 85%). Global cognitive functioning data were available for 62 individuals, while episodic memory (immediate and delayed recall) and semantic fluency data were available for 80 individuals.

### Leisure-time physical activity

Participants reported their LTPA behavior at all survey time points by using the structured and validated questions on commuting and LTPA (Waller et al., 2008). In these questions, participants reported their monthly frequency, duration, and intensity of LTPA, including active commuting to and from work, at mean ages of 45, 52, and 59 (we assumed there is no commuting activity in nonagenarians). Active commuting was included in LTPA because it is also voluntary and seems to have similar associations with health as LTPA (Schäfer et al., 2020). The LTPA variable we use in this study has been shown to associate with lower mortality and morbidity in earlier large population-based studies (Kujala et al., 1998; Waller et al., 2010). Earlier analyses have shown high correlations between the LTPA items used in the present study and physical activity data obtained by interview (Waller et al., 2008). Moreover, a detailed assessment of LTPA volume over the previous 12 months (12-month MET index) and a questionnaire-based LTPA MET index showed a good correlation (*r* = 0.73, *p* < .001) when the assessment and the questionnaire were administered at the same time point (Leskinen et al., 2009). The overall questionnaire-based physical activity behavior expressed as MET hours/day (the same variable used in the current study) correlated significantly with the accelerometer-measured physical activity variables, also in nonagenarians (*r* > 0.33, *p *< .05) (Aaltonen et al., 2023). The LTPA questions were included in all survey questionnaires in the same form, except at the mean age of 59 in 1990 when only one question was used. This question measures combined information on the frequency, duration, and intensity of LTPA, including commuting activity. The exact questions and response options for the questions are given in Supplementary Material Section 1. The overall LTPA level that was used to create LTPA trajectories from midlife to the tenth decade of life was quantified as metabolic equivalent of task (MET) hours expended per day. The calculation of MET hours per day is described in detail in Supplementary Section 2.1.

Accelerometer-measured physical activity was assessed in a subsample of 91-year-old participants (*N *= 36). These participants used a hip-worn tri-axial accelerometer for seven days to monitor their daily physical activity; see Aaltonen et al. (2023) and Supplementary Section 2.2 for details.

### Cognitive measures

Cognition was assessed with a telephone interview at the mean age of 91 years. From this telephone interview, a measure of global cognitive function could be derived in two different ways. The modified Telephone Interview for Cognitive Status (TICS-m) is a 15-item test of cognitive function of four domains: orientation, memory, attention, and language (score 0–50) (Lindgrén et al., 2019). This measure includes a 10-word list learning task that taps into episodic memory. TICS-m3 (score 0–70) is the same measure as TICS-m except it has three learning trials of the 10-word list instead of only one and correlates well with in-person Consortium to Establish a Registry for Alzheimer’s Disease Neuropsychological Battery (CERAD-nb) (Aaltonen et al., 2024). Immediate recall of episodic memory was described as the total number of words recalled in three learning trials of the 10-word list (score 0–30, immediate recall). Delayed recall of episodic memory was described as the number of words recalled after a 5-min delay (score 0–10) via telephone, a measure that performs similarly as an in-person measure of episodic memory (Saari et al., 2026). During the telephone interview after the TICS-m3, the participants were asked to list as many animals as they could in 1 min (score is the number of animals listed, describing semantic fluency).

### Covariates

The sex recorded at birth and date of birth were received from the Central Population Registry of Finland. We used the highest educational attainment (years of schooling) the participants had reported in 1975, 1981, or as nonagenarians as a measure of education.

### Ethics

The data collection was approved by the ethics committees of the Hjelt Institute, University of Helsinki, and the Helsinki and Uusimaa Hospital District, Finland. The latest ethical approval was given by the Ethics Committee of the Hospital District of Helsinki and Uusimaa for the NONAGINTA study protocol on May 8, 2020, and December 16, 2020 (the latest follow-up time point in the current study). All study methods were carried out in accordance with the approved guidelines and the Helsinki Declaration. All participants provided their written informed consents for the study by mail.

### Statistical analyses

#### Data-driven longitudinal leisure-time physical activity trajectories analysis

Because most trajectory modeling methods require a large sample size, we employed a more flexible method in terms of sample size. We used K-means cluster modeling for longitudinal data (KmL) to create and identify LTPA trajectories over a 46-year follow-up period with a total of up to four repeated measurements of physical activity (Genolini & Falissard, 2011). KmL is an enhanced version of the widely used K-means clustering algorithm, specifically designed to analyze and group longitudinal data consisting of repeated observations over time. Because different clustering validation measures have different weaknesses (Liu et al., 2010) and no singular index is superior (Todeschini et al., 2024), we relied on several different clustering indices. The optimum number of trajectories was chosen based on Calinski–Harabasz (with three versions), Ray–Turi, and Davies–Bouldin indices, as well as the sizes of the identified subgroups and the clinical relevance and stability of the LTPA trajectories. Stability was assessed with 500 iterations. The number of clusters that yields the maximum index is preferred over other solutions, and vice versa for Davies–Bouldin. We prioritized the Davies–Bouldin index over the Calinski–Harabasz index because it has been shown to provide more reliable results (Todeschini et al., 2024). KmL for longitudinal data was performed with the KmL package in R version 4.3.3 and RStudio version 2023.12.1 Build 402 (Genolini & Falissard, 2011). More details of the trajectory analyses are described in Supplementary Section 2.3.

#### Longitudinal leisure-time physical activity trajectory characteristics and their associations with cognition

When LTPA trajectories were identified, we continued analyses by investigating the associations between LTPA trajectory membership and cognition characteristics using generalized estimating equations (GEE) models (the “geepack” package in RStudio, R version 1.3.9). The GEE models are described in detail in Supplementary Section 2.4.

#### Longitudinal leisure-time physical activity trajectory characteristics and their associations with nonagenarians’ accelerometer-measured physical activity

To validate self-reported LTPA, we investigated the associations between LTPA trajectory membership and accelerometer-measured physical activity data in a subsample of nonagenarian participants (Aaltonen et al., 2023) using GEE models. We compared individuals in each trajectory to individuals in the two other trajectories, and sex and age were used as covariates.

#### Sensitivity analyses

As sensitivity analyses, we compared the differences in study participants’ characteristics (sex and the length of education) between those who did and did not participate in the cognition assessment, using a *t*-test for continuous variables and χ^2^ test for categorical variables (adjusted for clustered twin data) (Stata statistical software version 19.5, StataCorp, College Station, Texas, USA). Furthermore, we did a sensitivity analysis to confirm that the LTPA trajectories obtained were not dependent on missing values. We repeated the clustering by removing those participants who had missing values (R version 4.3.3 and RStudio version 2023.12.1 Build 402). We further compared the differences in the length of education between the LTPA trajectories and MET-hours per day between each time point using analysis of variance (ANOVA; Stata statistical software version 19.5, StataCorp, College Station, Texas, USA). The sensitivity of different versions of TICS to detect physical activity—cognition associations was compared (we did analyses separately for TICS-m and TICS-m3; no formal test was used for comparison).

## Results

The mean levels of LTPA among the full study cohort were 2.1 (standard deviation [*SD*] 1.8) MET-hours per day at age 45, 2.3 (*SD* 1.6) MET-hours per day at age 52, 2.4 (*SD* 1.9) MET-hours per day at age 59, and 1.6 (*SD* 1.7) MET-hours per day at age 91. The mean MET-hours per day were significantly higher at ages 52 and 59 compared to age 91 (*p *= .02 and *p *= .02, respectively).

### Longitudinal leisure-time physical activity trajectories

KmL analysis was specified to allow between two and six clusters (group-based trajectories), each obtained with 500 iterations. The optimum number of clusters was chosen based on several clustering validation indices and the general rule of parsimony (between two cluster solutions of equal value according to the clustering validation indices, we chose the one with less clusters, i.e., three cluster solution). The second iteration of three cluster solutions was chosen because of a lower Davies–Bouldin index and more balanced-sized clusters. Stability of the clusters was tested by repeating the analysis excluding the participants with missing values: the shapes of the three LTPA trajectories remained similar (Supplementary Figure 3). Clustering validation indices are reported in Supplementary Table 1 and Supplementary Figure 4, and six examples of clustering solutions are described in Supplementary Figure 5.

**Figure 1:**
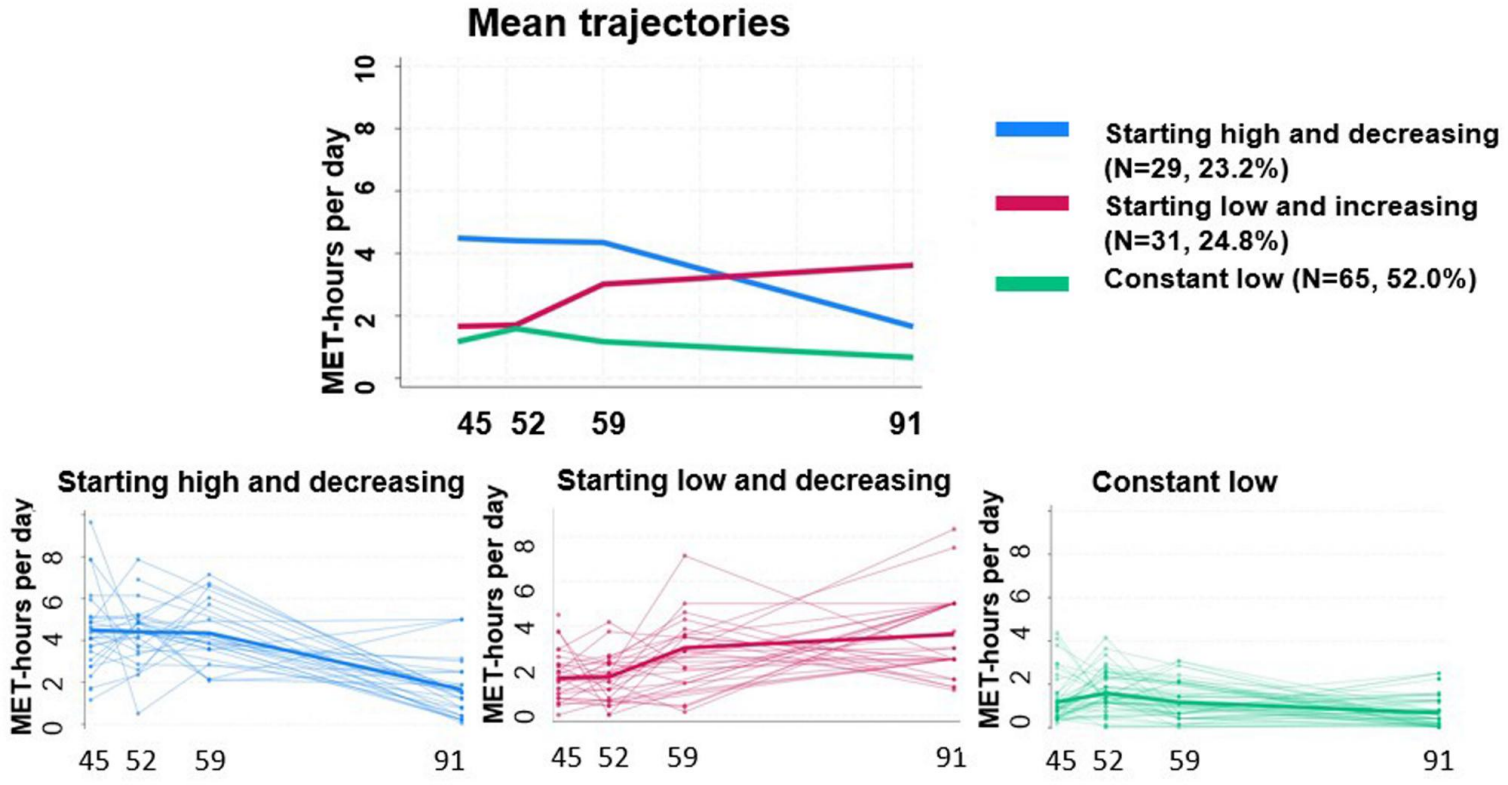
Leisure-time physical activity trajectories from midlife to the tenth decade of life with the spaghetti plots of individual trajectories. *Note*. LTPA = leisure-time physical activity.

We labeled the three distinct longitudinal LTPA trajectories identified by their unique elements as: *Constant low* (52.0%), *Starting low and increasing* (24.8%), and *Starting high and decreasing* (23.2%; Table 1 and Figure 1). The largest proportion of participants belonged to the *Constant low* trajectory. This trajectory was characterized by low LTPA throughout the follow-up, with a trend of a slight decrease: the mean leisure-time MET-hours per day were 1.2 (*SD* 1.0), 1.6 (*SD* 0.9), 1.2 (*SD* 0.9), and 0.7 (*SD* 0.7) at the mean ages of 45, 52, 59, and 91 years, respectively. These values approximately correspond to a daily 30-min walk at a slow pace in midlife and a daily 15-min walk at a slow pace at the mean age of 91.

**Table 1:** Characteristics of the study cohort by longitudinal leisure-time physical activity trajectories.

Characteristic variables	LTPA trajectories					
	Constant low		Starting high and decreasing		Starting low and increasing	
	*n* (%)	*M* (*SD*)	*n* (%)	*M* (*SD*)	*n* (%)	*M* (*SD*)
**Number of study participants**	65 (52.0)		29 (23.2)		31 (24.8)	
**Women**	45 (69.2)		18 (62.1)		18 (58.1)	
Years of education*		7.5 (3.1)		10.2 (4.9)		8.4 (4.2)
**Financial situation**		2.6 (0.6)		2.5 (0.5)		2.6 (0.6)
**Has someone to confide to**	51 (78.5)		22 (75.9)		23 (74.2)	
**Coronary artery disease**	18 (31.6)		5 (19.2)		12 (44.4)	
**Heart failure**	22 (39.3)		6 (24.0)		12 (41.4)	
**Stroke or transient ischemic attack**	16 (27.6)		3 (11.5)		6 (21.4)	
**Head trauma**	4 (7.3)		1 (3.4)		3 (10.3)	
**Malignancy**	13 (23.2)		9 (34.6)		4 (13.8)	
**Depression**	6 (10.9)		3 (11.1)		1 (3.5)	
**MET-hours per day age 45**		1.2 (1.0)		4.5 (1.9)		1.7 (1.0)
**MET-hours per day age 52**		1.6 (0.9)		4.4 (1.4)		1.7 (1.1)
**MET-hours per day age 59**		1.2 (0.9)		4.4 (2.1)		3.0 (1.8)
**MET-hours per day age 91**		0.7 (0.7)		1.7 (1.4)		3.6 (1.8)

*Note.* LTPA = leisure-time physical activity; *M* = mean; MET = metabolic equivalent of energy expenditure; *SD* = standard deviation. The characteristics between LTPA trajectories were compared with a χ^2^ test for categorical variables (adjusted for clustered twin data) and with a nonparametrical Kruskal–Wallis test for education. Statistically significant differences between LTPA trajectories are marked with an asterisk. Financial situation is reported on 1 to 5 scale (1 = very good, 2 = good, 3 = moderate, 4 = poor, 5 = very poor). Financial situation, social support, and comorbidities are self-reported (current or history of).

* *p *= .009 (The trajectory *Starting high and decreasing* was significantly different from the *Constant low* and *Starting low and increasing* trajectories).

The second largest proportion of participants belonged to the LTPA trajectory labeled as *Starting low and increasing,* and was characterized by a low level of LTPA at ages 45 and 52, with a clear increasing trend when approaching retirement age and the tenth decade of life (Table 1). The participants in this trajectory had a modest level of LTPA in their midlife but a moderate level in their sixties and nineties, approximately corresponding to a 30-min daily walk at a slow pace and a 40-min brisk daily walk, respectively.

The fewest participants were assigned to the *Starting high and decreasing* trajectory, which was characterized by the highest level of LTPA throughout the midlife years (i.e., working ages 45–59) with a decreasing trend from age 59 to age 91 (Table 1). In practice, the participants in the *Starting high and decreasing* trajectory had a 1-h brisk daily walk in their forties and fifties, while as nonagenarians, their LTPA corresponded to about a 30-min daily walk at a slow pace.

The study participants from the different LTPA trajectories differed statistically significantly only in terms of education, but not in terms of financial situation, social support, or comorbidities. The study participants who belonged to the Constant low trajectory had the lowest level of education, while the study participants belonging to the Starting high and decreasing trajectory had the highest education level on average. Our sensitivity analyses also indicated that the individuals who participated in the cognition assessments as nonagenarians were more educated than non-participants (*p *< .001; see Supplementary Table 2).

### Longitudinal leisure-time physical activity trajectory associations with cognition

**Figure 2:**
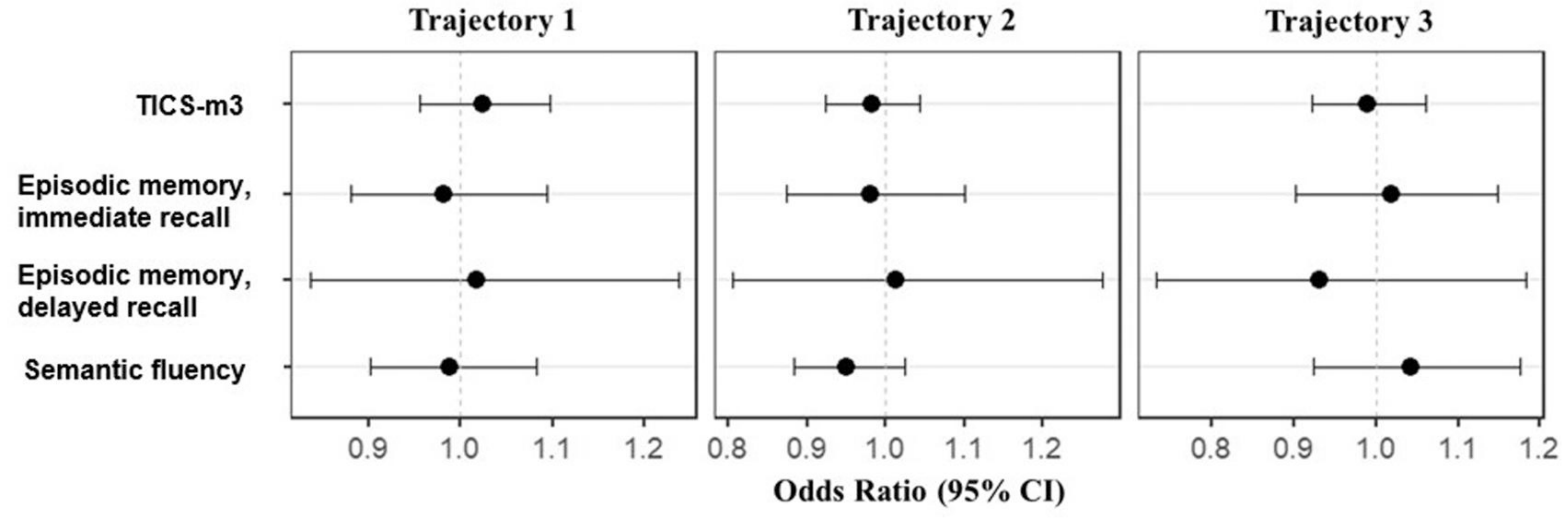
Summary statistics of cognition at age 91 by longitudinal leisure-time physical activity trajectories when adjusted for age, sex, and education. *Note*. CI = confidence interval; TICS = telephone interview for cognitive status; vs = versus. Participants from one leisure-time physical activity trajectory were compared to all participants from other trajectories.

**Figure 3:**
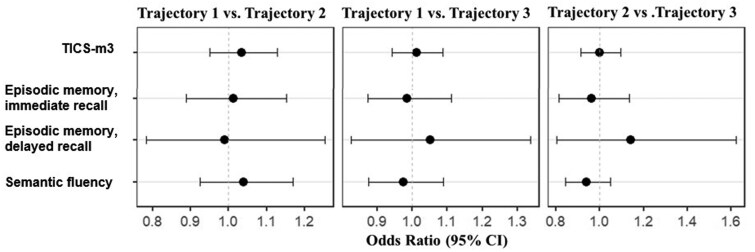
Summary statistics of pairwise comparisons of cognition at age 91 between each longitudinal leisure-time physical activity trajectory when adjusted for age, sex, and education. *Note*. CI = confidence interval; TICS = telephone interview for cognitive status; vs = versus.

First, we examined whether participants’ cognition in each LTPA trajectory was different from the cognition of the participants from other LTPA trajectories (one trajectory vs other trajectories). These analyses indicated no significant differences in cognition characteristics between trajectories (nominal *p *≥ .30) when adjusted for age and sex. The second set of analyses dealt with the pairwise comparisons between trajectories (one trajectory vs another trajectory), and again, we found no significant differences in cognition characteristics between LTPA trajectories when adjusted for sex and age (nominal *p *≥ .30). Both sets of analyses (i.e., [a] one trajectory vs other trajectories and [b] one trajectory vs another trajectory) were conducted again with sex, age, and education treated as covariates. We found no significant differences in cognition in these fully adjusted analyses (nominal *p *≥ .18 when one trajectory was compared to other trajectories and nominal *p *≥ .261 when one trajectory was compared to another trajectory) (Figures 2 and 3 and Supplementary Tables 3–6). The results did not differ when using differently modified versions of TICS (TICS-m and TICS-m3; Supplementary Table 3–6).

### Longitudinal leisure-time physical activity trajectory associations with nonagenarians’ accelerometer-measured physical activity

The lowest LTPA trajectory (*Constant low*) was associated with the lowest mean number of steps as well as the lowest average times of light and moderate-to-vigorous physical activity per day at the nonagenarian age when sex and age were taken into account (odds ratios between 0.22–0.43, nominal *p *< .048). Nonagenarians’ accelerometer-measured mean number of steps and the average times of light and moderate-to-vigorous physical activity per day in each trajectory are shown in Table 2.

**Table 2:** The associations between longitudinal leisure-time physical activity trajectories and nonagenarians’ accelerometer-measured physical activity.

LTPA trajectories	Nonagenarians’ accelerometer-measured physical activity		
	Mean low PA time per day hh:mm:ss (*SD*)	Mean moderate-to-vigorous PA time per day hh:mm:ss (*SD*)	Mean number of steps per day (*SD*)
**Constant low**	1:19:33^a^ (0:44:39)	0:05:33^a^ (0:05:46)	2,030^a^ (1,556)
**Starting low and increasing**	2:06:27 (0:45:10)	0:17:28 (0:20:44)	4,183 (3,133)
**Starting high and decreasing**	1:49:16 (0:44:22)	0:15:07 (0:19:41)	3,495 (2,761)

*Note*. h = hour; LTPA = leisure-time physical activity; m = minute; PA = physical activity; s = second; *SD* = standard deviation.

a Statistically significant differences between LTPA trajectories (the trajectory *Constant Low* was significantly different from the other two trajectories).

## Discussion

To our knowledge, our study is the first to generate nearly-five-decades-long LTPA trajectories from midlife to the tenth decade of life. We found three different LTPA trajectories. The *Constant low* trajectory was characterized by a low stable level of LTPA throughout the follow-up from midlife to the mean age of 91. The *Starting low and increasing* trajectory was also characterized by low levels of LTPA in midlife, but the participants in this trajectory first increased their LTPA level when they approached retirement age and as nonagenarians. The third LTPA trajectory, labeled *Starting high and decreasing,* portrayed a group of working age (ages 45, 52, and 59) individuals with moderate LTPA levels, but whose LTPA level notably decreased by the age of 91. Our results also revealed that cognition in nonagenarians was not associated with the LTPA trajectories identified. In our large worldwide meta-analytic dose-response analysis of physical activity and all-cause dementia combining data from 42 studies, the mean MET-hours per day was 2.1 (data published in Iso-Markku et al., 2022a), which is highly comparable to mean physical activity levels in this study population at ages 45, 52, and 59 (2.1, 2.3, and 2.4, respectively).

Previous studies on the association between longitudinal physical activity trajectories and cognition have used various methods to identify longitudinal trajectories of physical activity, including growth mixture modeling (Cheval et al., 2021; Hu et al., 2022), mixed models (Sabia et al., 2017), latent process mixed models (Wagner et al., 2020), and group-based trajectory modeling (Gafni et al., 2022; Gong & Baek, 2025; Li et al., 2022; Wang et al., 2024). The longitudinal physical activity trajectories found in these studies are very diverse, and the number of trajectories varies from two to five. A common feature for almost all studies presenting trajectories for absolute physical activity level is that they find one physical activity trajectory with a low stable pattern, and the largest proportion of participants belong to this trajectory (Gafni et al., 2022; Li et al., 2022; Wang et al., 2024). Of eight studies identifying physical activity trajectories and examining their associations with cognition, three have found a physical activity trajectory with a constant high (Cheval et al., 2021; Gafni et al., 2022; Li et al., 2022) and three with inconsistently high physical activity levels (Gafni et al., 2022; Hu et al., 2022; Wang et al., 2024). Two studies have used a case–control approach, showing either lower physical activity levels in cases throughout the whole follow-up (Wagner et al., 2020) or diverging physical activity trajectories approximately nine years before dementia diagnosis between cases and controls (Sabia et al., 2017). Our current results are consistent with these earlier studies, as we found one large, constantly low LTPA trajectory, and other LTPA trajectories had inconsistent physical activity levels. Our data-driven trajectory modeling provided us with one LTPA trajectory in which the highest LTPA levels were reached at ages 59 and 91. Similar increasing trajectories to ours have also been found in a few studies using model-based trajectory analysis methods (Hu et al., 2022; Li et al., 2022).

We did not find any significant association between nearly-five-decades-long LTPA trajectories and cognition in this small and selective cohort of nonagenarians when age, sex, and education were considered. An association was not even found among the participants of the *Starting high and decreasing* trajectory, despite some previous studies having found that the decreasing physical activity trajectories seem to precede cognitive decline (Hu et al., 2022) and dementia (Sabia et al., 2017). Conversely, our finding is consistent with earlier studies on physical activity and cognition in nonagenarians showing no association between these two (Gao et al., 2017; Gow et al., 2017; Strozza et al., 2020). Most earlier studies have also found either low or decreasing physical activity trajectories preceding cognitive decline (Cheval et al., 2021; Hu et al., 2022; Li et al., 2022; Sabia et al., 2017; Wagner et al., 2020; Wang et al., 2024), but these studies have assessed cognition mostly in individuals aged 65–80 years. Thus, it seems that a relationship between physical activity and cognition may be stronger earlier in life and weakens in older adults aged 85 years and over. We have looked at the association of physical activity separately at each time point of 1975, 1981, 1990, and at 90-year old, in association with cognition at nonagenarian age. In line with our current trajectory approach, these results indicated that LTPA measured in midlife or in old age was not associated with cognition at 90-year old (Varjonen et al., 2025). Our finding is in accordance with the study from Gong and Baek (2025), who similarly found that aging attenuates the positive association between physical activity trajectories and cognition. Our earlier meta-analysis showed no moderation by age on the association between physical activity and cognition, but the proportion of studies with nonagenarians included in the meta-analysis was small (Iso-Markku et al., 2024). Randomized controlled physical activity interventions to examine cognitive effects among specifically nonagenarians are practically lacking (Singh et al., 2025).

Our results suggest that other factors may be more influential in determining cognition than longitudinal LTPA behavior in exceptionally long-lived nonagenarians. This finding is in line with our study showing a weak association between cardiovascular risk factors and cognition in nonagenarians but indicating that higher education is associated with better cognition even when controlling for other risk factors (Varjonen et al., 2025) and with other studies showing no association between cardiovascular risk factors and dementia among older adults aged 85 years and over (Corrada et al., 2010). Previous studies have revealed that the cognitive performance of individuals aged 85 years and over has improved in more recent age cohorts compared to older age cohorts (Christensen et al., 2013; Schrijvers et al., 2012). Reasons for this shift seem to be the generally improved medical care (Schrijvers et al., 2012) and the so-called Flynn effect (i.e., cohorts born later cognitively outperform cohorts born earlier) (Christensen et al., 2013). It may be that the uneven distribution of unmeasured factors (e.g., the quality of medical care) or the general improvement in living standards from earlier times to current times contributes more to nonagenarian cognition than physical activity alone. At the same time, work-related physical activity has decreased substantially in the past five decades, which may represent an unmeasured confounder.

In our study, the participants in the *Constant low* trajectory had significantly lower levels of education than the participants in other trajectories. This finding is common (Cheval et al., 2021; Gafni et al., 2022; Gong & Baek, 2025; Li et al., 2022; Sabia et al., 2017; Wang et al., 2024). Earlier evidence shows that cognitive reserve, for which education serves as a proxy, moderates the association between lifestyles and cognitive aging so that cognitive reserve protects against deteriorative effects of unfavorable lifestyles (Franz et al., 2021; Iso-Markku et al., 2022a). The lack of an association between LTPA trajectories and cognition in our nonagenarians may reflect this phenomenon: those who survive to nonagenarian age are selected in terms of education, and in these individuals with a higher level of education, the association between lifestyles and cognition is weaker.

The spectrum of neurodegenerative pathology in the brain differs according to age (Nelson et al., 2016). In nonagenarians, for example, limbic-predominant age-related TAR DNA-binding protein 43 (TDP-43) encephalopathy, brain arteriosclerosis, and vascular dementia explain a large proportion of neurodegeneration, while these pathologies are rarer among 60- to 80-year olds (Nelson et al., 2016). Alzheimer’s disease remains, however, the most prevailing neuropathology in both age groups. We have shown in our previous study that physical activity is associated with a lower incidence of both Alzheimer’s disease and vascular dementia (Iso-Markku et al., 2022b). Consequently, the varying spectrum of neurodegeneration across aging is an unlikely explanation for the lack of association between 46-year LTPA trajectories and cognition in nonagenarians.

Mechanisms via which physical activity may decelerate both pathological and aging-related cognitive decline are numerous: cerebral blood flow, hippocampal volume, neuroinflammation, mitochondrial function, autophagy and proteasomal degradation, epigenetic modifications, brain plasticity, neurogenesis, and microbiota both in the gut and oral cavity (Tari et al., 2025). Despite this vast plethora of possible mechanisms via which physical activity may be beneficial for brain health, many studies show no gain despite the “pain.” For example, a meta-analysis of exercise intervention studies shows no influence on brain volume (Gogniat et al., 2021), and large longitudinal observational studies show a reverse association: brain volume predicts physical activity participation instead of vice versa (Hofman et al., 2023; Rodriguez-Ayllon et al., 2024). Even though super-agers tend to be both physically and mentally strong (Hermansen et al., 2024), the conundrum of which comes first, the chicken or the egg, better cognition or physical activity, continues. It must also be remembered that understanding aging is not a simple matter, and associations between lifestyles and diseases, such as neurodegenerative conditions, are intertwined with many factors. For example, genetics are shown to affect both physical activity behavior (Lightfoot et al., 2018) and cognition (Polderman et al., 2015).

There are some limitations in our study that need to be considered. Although we constructed unique and exceptionally long LTPA trajectories, the greatest weakness of our study is the small sample size, which may inhibit us from detecting a weak association between LTPA trajectories and cognition. In our population-based study, all twin individuals (a) who were the members of same-sexed twin pairs born in Finland before 1958, (b) who reported their LTPA behavior in two survey questionnaires at least during the 46-year follow-up, and (c) who participated in telephone-interviews as nonagenarians were included in the study. This is a rare study design, and thus, not very large sample size can be expected. However, it is important to note that the small sample size can lead to low statistical power, can increase the risk of random errors, and may inhibit us from detecting a weak association between LTPA trajectories and cognition.

Additionally, the follow-up time between the last two time points was over 30 years: shorter intervals would have provided more precise data on LTPA patterns. It is also possible that our LTPA results are biased, given the self-reported nature of LTPA. However, the self-reported LTPA items and accelerometer-measured physical activity behavior we used in the current study have been shown to have high validity (Aaltonen et al., 2023; Leskinen et al., 2009; Waller et al., 2008). Finally, cognition was assessed with a telephone interview in the present study. The telephone interview is more vulnerable to interference due to hearing problems. Individuals with mild-to moderate hearing problems have stated, however, that they heard better via telephone than face-to-face, and an earlier study of this cohort has shown that the overall performance in the telephone cognition interview did not differ significantly between those using and not using hearing aids (Lindgrén, 2021). Unfortunately, we did not have information on cognitive abilities from the beginning of the follow-up. This is a major limitation, since early cognitive ability has been shown to predict later physical activity participation and to explain in part physical activity–cognition associations (Iso-Markku et al., 2025).

Due to missing data and differential participation across waves, our nonagenarian participants are selected in terms of education (8.4 years of education vs 8.0 at a mean age 72 [Iso-Markku et al., 2021]) and most likely in terms of other attributes as well. For example, substance use is a common, known factor for missing data in research. Compared to many study cohorts, our study cohort is still exceptionally unselected; for example, the formal education level is still exceptionally low in the population-based Older Finnish Twin Cohort. Only a small fraction of school children would go on to secondary or tertiary education in the early postwar years after the Continuation War (1941–1944). In addition, mortality and dementia share risk factors, including APOE genotype (Deelen et al., 2019; Kunkle et al., 2019) and education (Meng & D’Arcy, 2012; Ye et al., 2023). Thus, survivorship bias may also hinder us from detecting LTPA-induced differences in nonagenarians’ cognition.

The main strength of this study is the 46-year follow-up of LTPA. As far as we know, our current study examining prior lifestyle habits extending from age 45 to almost five decades ahead is unprecedented. Overall, studies on the determinants of nonagenarians’ cognition are also very rare. We also assessed LTPA with many survey questions, allowing for more precise estimates of LTPA levels: the frequency, intensity, and duration of LTPA, including active commuting, were assessed at each follow-up time point, except at the last follow-up time point, when participants were 91-year old on average, and commuting activity was no longer relevant. An additional strength is that our population-based study cohort has quite an equal sex representation. Thus, our results may be more generalizable at the population level.

In conclusion, we provide new knowledge on longitudinal LTPA trajectories from midlife to the tenth decade of life and their association with nonagenarian cognition. Nearly five-decade-long LTPA trajectories were not significantly associated with nonagenarians’ cognition. Our results may also be partly explained by survivorship bias and a small sample size. Studies with a larger sample size on the topic could provide more definitive evidence in the future and are, thus, warranted. Moreover, as the number of nonagenarians continues to rise, more research should be directed to disentangling potential factors affecting cognition in older adults aged 85 years and over. Although we did not find significant associations between nearly-five-decades-long LTPA trajectories and nonagenarians’ cognition, LTPA is recommended due to its numerous other benefits for cardiovascular function, physical function, and mental health.

## Supplementary Material

gbag065_Supplementary_Data

## Data Availability

The data used in the study are not publicly available due to the restrictions of informed consent given by participants. However, the data can be requested through the Institute for Molecular Medicine Finland (FIMM) Data Access Committee (DAC; fimm-dac@helsinki.fi) for authorized researchers who have ethical approval and an institutionally approved study plan. To ensure the protection of privacy and compliance with national data protection legislation, a data use/transfer agreement is needed, the content and specific clauses of which will depend on the nature of the requested data. This study was not preregistered.
