# Supplementary material for: Data-driven leisure-time physical activity trajectories of over 46 years and their associations with cognition in nonagenarians: a cohort study
Source: J Gerontol B Psychol Sci Soc Sci. 2026 Apr 9;81(6):gbag065. doi: 10.1093/geronb/gbag065 (PMC13180642; doi:10.1093/geronb/gbag065)
Supplement: gbag065_Supplementary_Data [file gbag065_supplementary_data.pdf]

The Journals of Gerontology, Series B: Psychological Sciences and Social Sciences Supplementary Material: Data-Driven Leisure-Time Physical Activity Trajectories of Over 46 Years and Their Associations with Cognition in Nonagenarians: A Cohort Study.

Table of Contents

Section 1: Leisure-time physical activity questions 2

Section 2: Methods 4

Section 3: Supplementary Tables and Figures 7

Section 1: Leisure-time physical activity questions

Questions have been translated from Finnish.

Leisure-time physical activity question at the mean ages 45 and 52:

How much of your daily journey to work/study is spent in walking, cycling, running and/or cross-country skiing?
1 less than 15 min
2 15 min to less than half an hour
3 half an hour to less than one hour
4 one hour or more
5 I am presently not at work/studying

How often do you exercise/engage in physical activity during your leisure time?
1 less than once a month
2 1–2 times a month
3 3–5 times a month
4 6–10 times a month
5 11–19 times a month
6 more than 20 times a month

Is your physical activity during leisure time about as tiring on average as:
1 walking
2 alternatively walking and jogging
3 jogging (light run)
4 running

How long does one session of physical activity last on average?
1 less than 15 min
2 15 min to less than half an hour
3 half an hour to less than one hour
4 one hour to under two hours
5 two hours or more

Leisure-time physical activity question at mean age 59:

In the next question, we are going to inquire about your leisure-time and commuting physical activity during the last 12 months. We have divided physical activity into four different intensities. First, evaluate how strenuous each of the physical activities you engage in are. Then, evaluate, on average, how many hours per week you engage in physical activity corresponding to each intensity level?

Intensity level of each physical activity	Not at all	Altogether less than ½ hour per week	Altogether ½ – 1 hour per week	Altogether 2 – 3 hours per week	Altogether 4 hours or more per week
Walking	1	2	3	4	5
Alternatively walking and jogging	1	2	3	4	5
Jogging (light run)	1	2	3	4	5
Running	1	2	3	4	5

Leisure-time physical activity questions at mean age 91:

How often do you exercise/engage in physical activity sessions?

1 less than once a month
2 1–2 times a month
3 3–5 times a month
4 6–10 times a month
5 11–19 times a month
6 more than 20 times a month

Is your physical activity as tiring on average as:
1 walking
2 alternatively walking and jogging
3 jogging (light run)
4 running

How long does one session of physical activity last on average?
1 less than 15 min
2 15 min to less than half an hour
3 half an hour to less than one hour
4 one hour to under two hours
5 two hours or more

Section 2: Methods

1. Self-reported physical activity

To calculate MET hours per day from the LTPA questions, we used the following formula: LTPA frequency (average per day) × LTPA duration (average hours) × LTPA intensity (activity MET score) (+ average active commuting per day at mean ages 45, 52, 59). The following MET score values were used for the intensity of LTPA to obtain a multiple of the resting metabolic rate for each activity: 4 corresponded to walking, 6 corresponded to vigorous walking to jogging, 10 corresponded to jogging, and 13 corresponded to running. The MET value of 4 (walking) was also used for the intensity of commuting-related physical activity. We further assumed that commuting-related physical activity was done five days per week. All types of LTPAs were considered when MET hours per day were calculated.

2. Accelerometer-measured physical activity

Hip-worn tri-axial accelerometer (UKK RM42, UKK Terveyspalvelut Oy, Tampere, Finland) along with instructions were mailed to the study participants of the telephone interview of NONAGINTA study. The accelerometers were advised to be worn on right side of the hip with an elastic band for seven consecutive days during waking hours except for shower, swimming and bathing. Measurements with at least 10 hours' wear-time per day and worn on at least 4 days were included in the analyses. The acceleration data were recorded at a sampling frequency of 100 Hz. We used mean amplitude deviation (MAD) and angle for posture estimation (APE) algorithms in the analyses when calculating MET-hours from the raw data (1). MET-values were smoothed by calculating the moving average for each 6-second epoch.

3. Details of the trajectory analyses

Prior to trajectory analysis, we excluded 2 outliers with over 15 MET-hours per day (corresponds to about a 1.5-hour run at a pace of 10 kilometers per hour) when participants were at mean age 59 in 1990 and normalized the data using the min-max normalization method. We allowed for a maximum of two missing time points. The stability of the clusters was tested with 500 permutations for each cluster number, the validation indices and the sensitivity analysis in which we removed study participants with missing values.

4. Generalized estimating equations models

We utilized generalized estimating equation models to assess associations between LTPA trajectory membership and cognitive characteristics, accounting for within family dependence. Because observations may be correlated within twin pairs but not across pairs, we specified an exchangeable working correlation structure. We conducted two sets of analyses. In the first set of analyses, we compared individuals in each trajectory to those in the other two trajectories, while in the second set of analyses, we involved pairwise comparisons between the trajectories. In both sets of analyses, we modeled LTPA trajectories as outcomes and cognition characteristics as independent variables. Continuous cognitive measures were used as such in the analyses. Both sets of analyses were first conducted with sex and age as covariates, and then with sex, age, and education as covariates. We tested the null hypothesis that the coefficients of the cognitive variables were equal to zero and reported odds ratios along with their 95% confidence intervals and p-values. P-values were reported both as nominal and corrected for multiple testing within each set of

analyses using the Benjamini–Hochberg procedure. Listwise deletion was used to handle missing data.

References

Vähä-Ypyä, H., Vasankari, T., Husu, P., Suni, J., & Sievänen, H. (2015). A universal, accurate intensity-based classification of different physical activities using raw data of accelerometer. *Clinical Physiology and Functional Imaging*, 35(1), 64–70. https://doi.org/10.1111/cpf.12127

Section 3: Supplementary Tables and Figures

Supplementary Table 1. The comparison of six different clustering solutions in identifying 46-year-long leisure-time physical activity trajectories using K-mean cluster modeling.

Number of clusters	Calinski-Haribaszindex	Calinski-Harabaszindex 2	Calinski-Harabaszindex 3	Ray-Turi	Davies-Bouldin	Sizes of clusters
2	78.15186910	0.6405467492	78.15186910	-0.0887908130	-1.276220481	82;43
**3**	**69.14427808**	**1.152094931**	**97.78477582**	**-0.1085795173**	**-1.338537211**	**65;31;29**
3	69.16918789	1.152509983	97.82000361	-0.1105532049	-1.341208561	65;33;27
4	58.53578732	1.487283169	101.3869577	-0.1490310090	-1.370455004	61;26;25;13
5	52.23451244	1.799188761	104.4690248	-0.2823481970	-1.399690875	40;29;25;19;12
6	49.3854007	2.162202418	110.4291131	-0.2786909543	-1.457110478	39;29;18;15;12;12

Note. The final solution selected is in bold.

Supplementary Table 2. The comparison of study participants' characteristics between the participants and non-participants of cognition assessments.

	**Participated in cognition assessment** (n=80)	**Did not participate in cognition assessment** (n=45)	**P-value for difference between groups***
**Sex**			
Men (n, %)	33 (41.3)	14 (29.2)	
Women (n, %)	47 (58.5)	34 (70.8)	0.093
**Education years** (mean, SD)	9.4 (4.6)	6.6 (1.6)	< 0.001
**MET-hours per day age 45** (mean, SD)	2.2 (1.9)	1.9 (1.8)	0.388
**MET-hours per day age 52** (mean, SD)	2.5 (1.7)	2.0 (1.4)	0.165
**MET-hours per day age 59** (mean, SD)	2.6 (2.1)	2.2 (1.6)	0.384
**MET-hours per day age 91** (mean, SD)	1.7 (1.6)	1.6 (1.9)	0.849

* Difference between groups has been compared with t-test (adjusted for clustered twin data)

Abbreviations: MET, metabolic equivalent of energy expenditure; SD, standard deviation.

Supplementary Table 3. Differences in cognition by longitudinal LTPA trajectories. Participants from one LTPA trajectory were compared to all participants from other trajectories. The analyses were adjusted for age and sex.

Variable	Trajectory class	Mean, trajectory class	Mean, other trajectory classes	Odds ratio	CI		Cohen's d	CI		p-values	
					Lower bounds	Upper bounds		Lower bounds	Upper bounds	Nominal	Benjamini-Hochberg
Semantic fluency	1	14.56	15.59	0.96	0.87	1.04	0.03	-0.03	0.10	0.313	0.907
Semantic fluency	2	14.69	15.16	0.98	0.91	1.07	-0.01	-0.06	0.04	0.681	0.907
Semantic fluency	3	16.22	14.6	1.06	0.95	1.2	-0.02	-0.08	0.04	0.296	0.907
TICS-m3	1	37.07	39.33	0.98	0.92	1.04	0.01	-0.02	0.05	0.454	0.907
TICS-m3	2	39.07	38.04	1.01	0.95	1.08	-0.01	-0.05	0.02	0.723	0.907
TICS-m3	3	39.53	37.72	1.01	0.94	1.08	-0.01	-0.05	0.03	0.786	0.907
Episodic memory, immediate recall	1	11.37	12.21	0.93	0.83	1.04	-0.01	-0.07	0.05	0.198	0.907
Episodic memory, immediate recall	2	11.94	11.73	1.02	0.91	1.14	-0.01	-0.08	0.05	0.756	0.907
Episodic memory, immediate recall	3	12.39	11.53	1.05	0.94	1.18	0.01	-0.06	0.08	0.413	0.907
Episodic memory, delayed recall	1	2.24	2.56	0.94	0.78	1.14	0.01	-0.10	0.12	0.534	0.907
Episodic memory, delayed recall	2	2.62	2.34	1.08	0.85	1.36	0.01	-0.12	0.14	0.527	0.907
Episodic memory, delayed recall	3	2.52	2.35	1	0.82	1.23	-0.04	-0.17	0.09	0.975	0.993
TICS-m	1	28.1	29.48	0.98	0.91	1.06	-0.01	-0.06	0.04	0.663	0.907
TICS-m	2	29.43	28.67	1.02	0.92	1.12	-0.03	-0.07	0.01	0.720	0.907
TICS-m	3	29.53	28.53	1	0.91	1.1	0.02	-0.05	0.09	0.993	0.993

Note. Trajectory class 1 = Constant Low; Trajectory class 2 = Starting Low and Increasing; Trajectory class 3 = Starting High and Decreasing; LTPA, leisure-time physical activity; TICS-m =the total score of TICS-m after one-word list learning trial; TICS-m3 = the total score of TICS-m after three-word list learning trial

Supplementary Table 4. Pairwise comparisons of cognition between each longitudinal LTPA trajectory. The analyses were adjusted for age and sex.

Variable	Trajectory class A	Trajectory class B	Mean, trajectory class A	Mean, trajectory class B	Odds ratio	Confidence intervals		Cohen's d	Confidence intervals		p-values	
						Lower bound	Upper bound		Lower bound	Upper bound	Nominal	Benjamini-Hochberg
TICS-m3	1	2	37.07	39.07	0.98	0.90	1.06	-0.01	-0.06	0.03	0.569	0.964
TICS-m3	1	3	37.07	39.53	0.98	0.92	1.05	-0.01	-0.05	0.03	0.573	0.964
TICS-m3	2	3	39.07	39.53	1.00	0.92	1.08	0.00	-0.05	0.04	0.937	0.964
Episodic memory, immediate recall	1	2	11.37	11.94	0.96	0.84	1.09	-0.02	-0.10	0.05	0.481	0.964
Episodic memory, immediate recall	1	3	11.37	12.39	0.95	0.84	1.07	-0.03	-0.10	0.04	0.366	0.964
Episodic memory, immediate recall	2	3	11.94	12.39	0.97	0.84	1.12	-0.02	-0.10	0.06	0.646	0.964
Episodic memory, delayed recall	1	2	2.24	2.62	0.93	0.73	1.18	-0.04	-0.17	0.09	0.533	0.964
Episodic memory, delayed recall	1	3	2.24	2.52	0.99	0.79	1.23	-0.01	-0.13	0.11	0.910	0.964
Episodic memory, delayed recall	2	3	2.62	2.52	1.05	0.80	1.39	0.03	-0.12	0.18	0.714	0.964
Semantic fluency	1	2	14.56	14.69	0.99	0.89	1.10	-0.01	-0.06	0.05	0.822	0.964
Semantic fluency	1	3	14.56	16.22	0.94	0.85	1.05	-0.03	-0.09	0.03	0.300	0.964
Semantic fluency	2	3	14.69	16.22	0.94	0.84	1.05	-0.03	-0.10	0.03	0.295	0.964
TICS-m	1	2	28.10	29.43	0.98	0.86	1.10	-0.01	-0.08	0.05	0.701	0.964
TICS-m	1	3	28.10	29.53	0.99	0.90	1.09	-0.01	-0.06	0.05	0.878	0.964
TICS-m	2	3	29.43	29.53	1.00	0.90	1.12	0.00	-0.06	0.06	0.964	0.964

Note. Trajectory class 1 = Constant Low; Trajectory class 2 = Starting Low and Increasing; Trajectory class 3 = Starting High and Decreasing; LTPA, leisure-time physical activity; TICS-m =the total score of TICS-m after one-word list learning trial; TICS-m3 = the total score of TICS-m after three-word list learning trial.

Supplementary Table 5. Differences in cognition by longitudinal LTPA trajectory. Participants from one LTPA trajectory were compared to all participants from other trajectories. The analyses were adjusted for age, sex, and education.

Variable	Trajectory class	Mean, trajectory class	Mean, other trajectory classes	Odds ratio	Confidence intervals		Cohen's d	Confidence intervals		p-values	
					Lower bound	Upper bound		Lower bound	Upper bound	Nominal	Benjamini-Hochberg
TICS-m3	1	37.07	39.33	1.02	0.96	1.10	0.01	-0.02	0.05	0.493	0.903
TICS-m3	2	39.07	38.04	0.98	0.92	1.04	-0.01	-0.05	0.02	0.565	0.903
TICS-m3	3	39.53	37.72	0.99	0.92	1.06	-0.01	-0.05	0.03	0.756	0.903
Episodic memory, immediate recall	1	11.37	12.21	0.98	0.88	1.09	-0.01	-0.07	0.05	0.737	0.903
Episodic memory, immediate recall	2	11.94	11.73	0.98	0.87	1.10	-0.01	-0.08	0.05	0.737	0.903
Episodic memory, immediate recall	3	12.39	11.53	1.02	0.90	1.15	0.01	-0.06	0.08	0.776	0.903
Episodic memory, delayed recall	1	2.24	2.56	1.02	0.84	1.24	0.01	-0.10	0.12	0.861	0.903
Episodic memory, delayed recall	2	2.62	2.34	1.01	0.81	1.28	0.01	-0.12	0.14	0.903	0.903
Episodic memory, delayed recall	3	2.52	2.35	0.93	0.73	1.18	-0.04	-0.17	0.09	0.554	0.903
Semantic fluency	1	14.56	15.59	0.99	0.90	1.08	-0.01	-0.06	0.04	0.790	0.903
Semantic fluency	2	14.69	15.16	0.95	0.88	1.02	-0.03	-0.07	0.01	0.184	0.903
Semantic fluency	3	16.22	14.60	1.04	0.92	1.18	0.02	-0.05	0.09	0.500	0.903
TICS-m	1	28.10	29.48	1.06	0.95	1.19	0.03	-0.03	0.10	0.268	0.903
TICS-m	2	29.43	28.67	0.98	0.90	1.07	-0.01	-0.06	0.04	0.635	0.903
TICS-m	3	29.53	28.53	0.97	0.87	1.07	-0.02	-0.08	0.04	0.477	0.903

Note. Trajectory class 1 = Constant Low; Trajectory class 2 = Starting Low and Increasing; Trajectory class 3 = Starting High and Decreasing; LTPA, leisure-time physical activity; TICS-m =the total score of TICS-m after one-word list learning trial; TICS-m3 = the total score of TICS-m after three-word list learning trial

Supplementary Table 6. Pairwise comparisons of cognition between each longitudinal LTPA trajectory. The analyses were adjusted for age, sex, and education.

Variable	Trajectory class A	Trajectory class B	Mean, trajectory class A	Mean, trajectory class B	Odds ratio	Confidence intervals		Cohen's d	Confidence intervals		p-values	
						Lower bound	Upper bound		Lower bound	Upper bound	Nominal	Benjamini-Hochberg
TICS-m3	1	2	37.07	39.07	1.03	0.95	1.13	0.02	-0.03	0.07	0.439	0.993
TICS-m3	1	3	37.07	39.53	1.01	0.94	1.09	0.01	-0.03	0.05	0.734	0.993
TICS-m3	2	3	39.07	39.53	1.00	0.91	1.10	0.00	-0.05	0.05	0.993	0.994
Episodic memory, immediate recall	1	2	11.37	11.94	1.01	0.89	1.15	0.01	-0.06	0.08	0.860	0.993
Episodic memory, immediate recall	1	3	11.37	12.39	0.99	0.87	1.11	-0.01	-0.08	0.06	0.807	0.993
Episodic memory, immediate recall	2	3	11.94	12.39	0.96	0.81	1.13	-0.02	-0.12	0.07	0.634	0.993
Episodic memory, delayed recall	1	2	2.24	2.62	0.99	0.78	1.25	-0.01	-0.14	0.12	0.936	0.994
Episodic memory, delayed recall	1	3	2.24	2.52	1.05	0.83	1.34	0.03	-0.10	0.16	0.684	0.993
Episodic memory, delayed recall	2	3	2.62	2.52	1.14	0.80	1.62	0.07	-0.12	0.27	0.462	0.993
Semantic fluency	1	2	14.56	14.69	1.04	0.92	1.17	0.02	-0.05	0.09	0.519	0.993
Semantic fluency	1	3	14.56	16.22	0.98	0.88	1.09	-0.01	-0.07	0.05	0.662	0.993
Semantic fluency	2	3	14.69	16.22	0.94	0.84	1.05	-0.03	-0.10	0.03	0.261	0.993
TICS-m	1	2	28.10	29.43	1.08	0.94	1.24	0.04	-0.03	0.12	0.295	0.993
TICS-m	1	3	28.10	29.53	1.05	0.94	1.17	0.03	-0.03	0.09	0.428	0.993
TICS-m	2	3	29.43	29.53	1.01	0.90	1.13	0.01	-0.06	0.07	0.847	0.993

Note. Trajectory class 1 = Constant Low; Trajectory class 2 = Starting Low and Increasing; Trajectory class 3 = Starting High and Decreasing; LTPA; leisure-time physical activity; TICS-m =the total score of TICS-m after one-word list learning trial; TICS-m3 = the total score of TICS-m after three-word list learning trial.

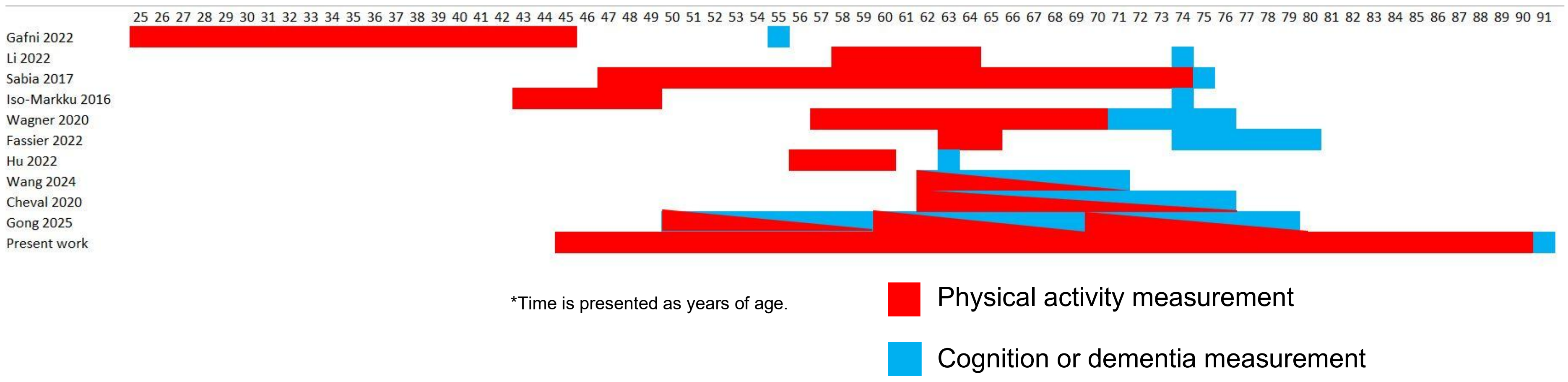

Supplementary Figure 1. Timeline of observational studies assessing the association of longitudinal physical activity (measured at multiple time points) and cognition

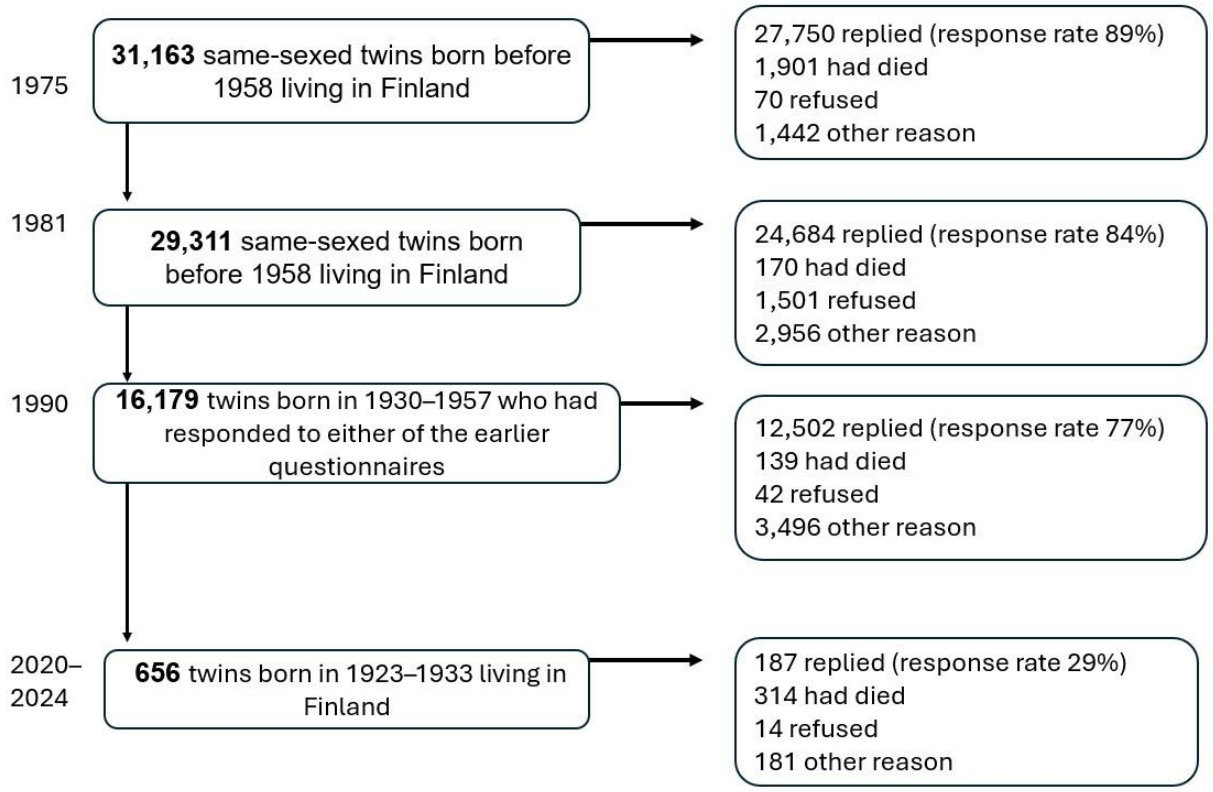

Supplementary Figure 2. Flowchart of the study

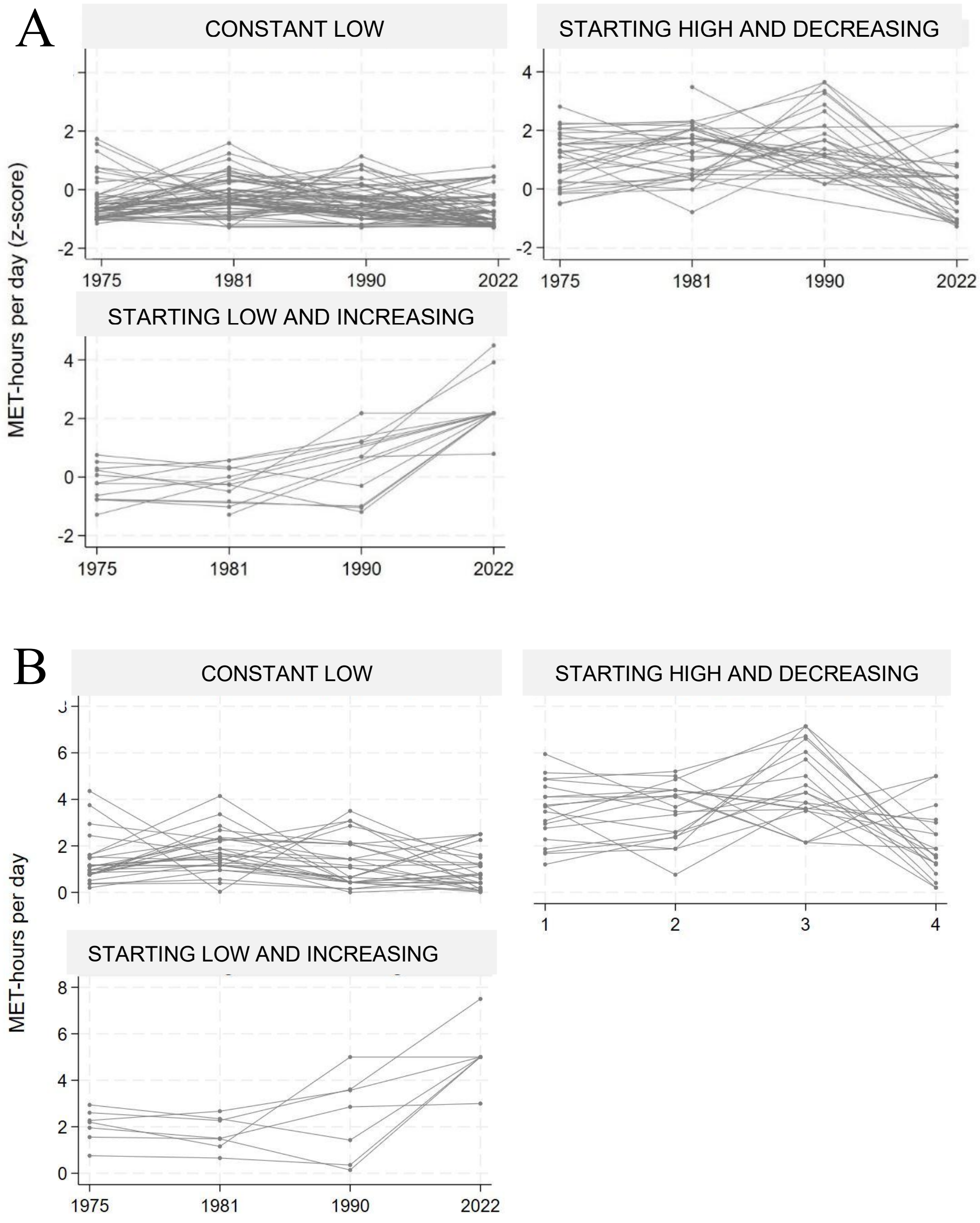

Supplementary Figure 3. Sensitivity analysis by excluding the individuals with missing time points. A) The LTPA trajectories of the chosen clustering solution. B) The LTPA trajectories of the chosen clustering solution with only individuals without any missing time points.

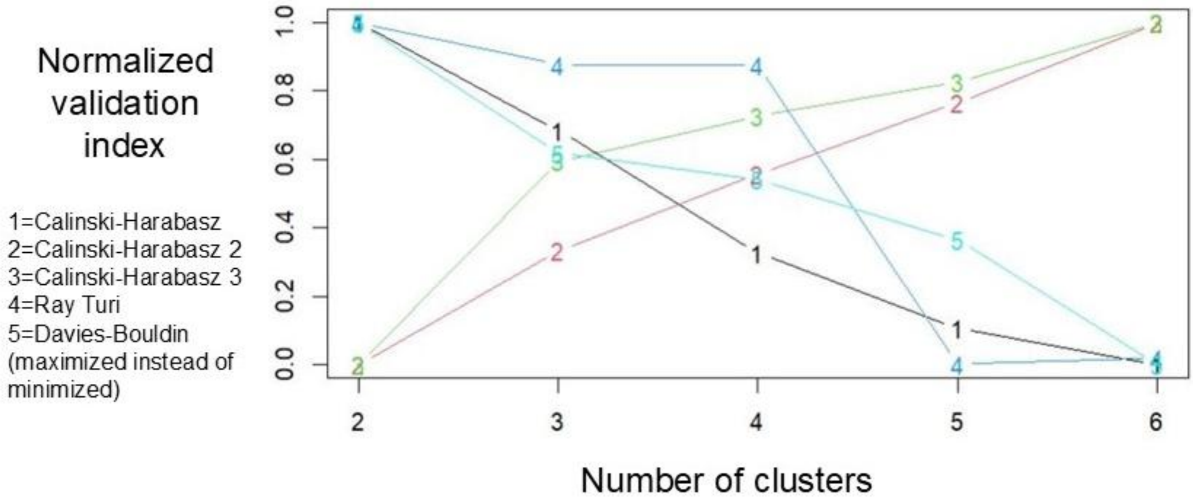

Supplementary Figure 4. Validation indices in identifying leisure-time physical activity trajectories using K-mean cluster modeling

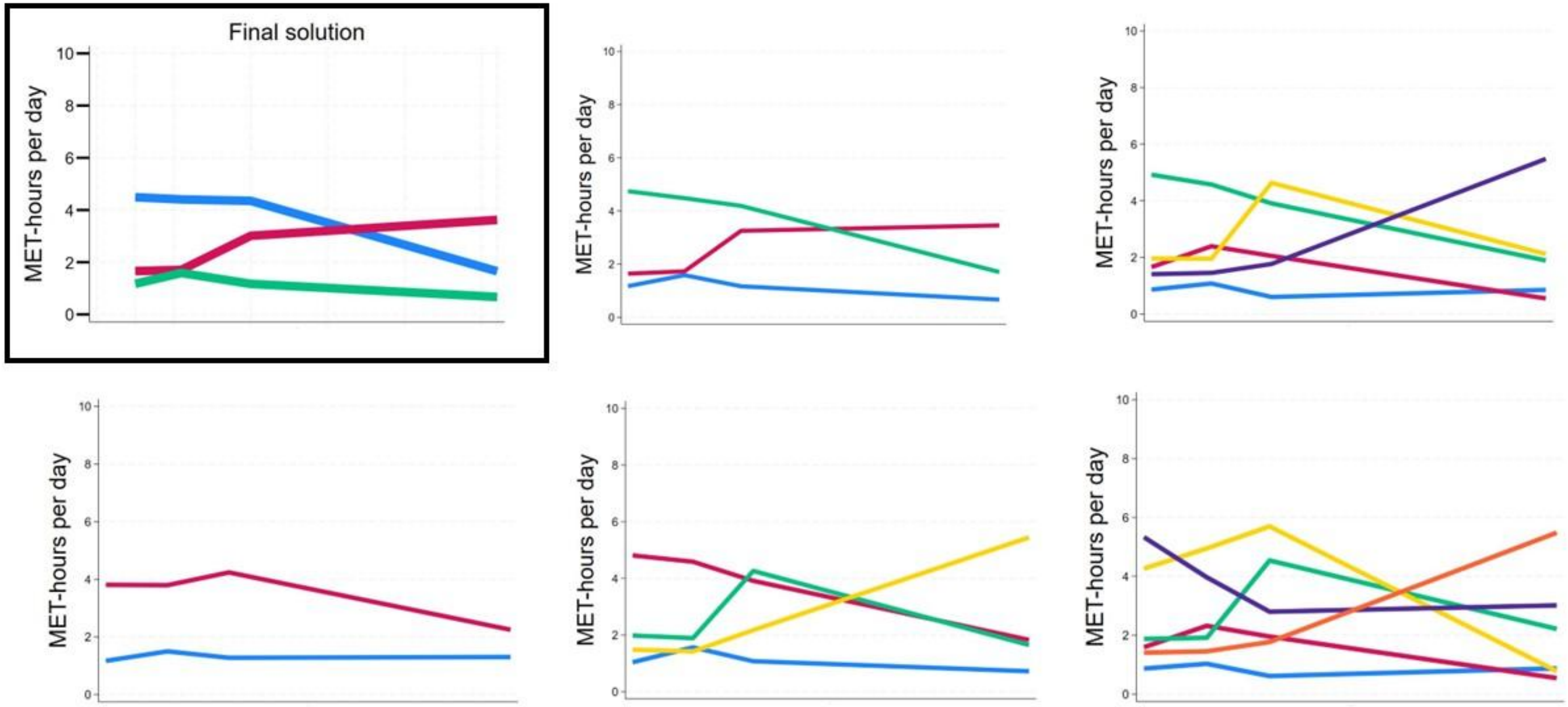

Supplementary Figure 5. Six examples of clustering solutions in identifying leisure-time physical activity trajectories using K-mean cluster modeling
